# A genetic association analysis of cognitive ability and cognitive ageing using 325 markers for 109 genes associated with oxidative stress or cognition

**DOI:** 10.1186/1471-2156-8-43

**Published:** 2007-07-02

**Authors:** Sarah E Harris, Helen Fox, Alan F Wright, Caroline Hayward, John M Starr, Lawrence J Whalley, Ian J Deary

**Affiliations:** 1 Department of Psychology, University of Edinburgh, 7 George Square, Edinburgh EH8 9JZ, UK; 2 Department of Mental Health, University of Aberdeen, Clinical Research Centre, Royal Cornhill Hospital, Cornhill Road, Aberdeen, AB25 2ZH, UK; 3 Medical Research Council Human Genetics Unit, Western General Hospital, Crewe Road, Edinburgh EH4 2XU, UK; 4 Department of Geriatric Medicine, University of Edinburgh, Royal Victoria Hospital, Craigleith Road, Edinburgh EH4 2DN, UK

## Abstract

**Background:**

Non-pathological cognitive ageing is a distressing condition affecting an increasing number of people in our 'ageing society'. Oxidative stress is hypothesised to have a major role in cellular ageing, including brain ageing.

**Results:**

Associations between cognitive ageing and 325 single nucleotide polymorphisms (SNPs), located in 109 genes implicated in oxidative stress and/or cognition, were examined in a unique cohort of relatively healthy older people, on whom we have cognitive ability scores at ages 11 and 79 years (LBC1921). SNPs showing a significant positive association were then genotyped in a second cohort for whom we have cognitive ability scores at the ages of 11 and 64 years (ABC1936). An intronic SNP in the *APP *gene (rs2830102) was significantly associated with cognitive ageing in both LBC1921 and a combined LBC1921/ABC1936 analysis (*p *< 0.01), but not in ABC1936 alone.

**Conclusion:**

This study suggests a possible role for APP in normal cognitive ageing, in addition to its role in Alzheimer's disease.

## Background

Individuals differ in their cognitive skills, and in how much these cognitive skills change as people grow older. That is, there are individual differences in the trait (or level) of intelligence, and in the age-related change (or trajectory). We have previously shown that about 50% of the variance in trait intelligence is stable from the age of 11 to the age of 79[1]. In both the trait and the age-related change, the majority of the between-individual variation is accounted for by a common factor of general cognitive ability (or *g*)[2,3]. Both mild intellectual impairment (low trait intelligence) and accelerated age-related cognitive decline (increased downward trajectory in intelligence) have a major impact on society, because of the large number of individuals involved who have limited independence. In our increasingly 'ageing society', disabilities linked to cognitive ageing are a growing medical and social problem.

There are environmental and genetic contributions to individual differences in trait intelligence and cognitive ageing[4,5]. Genetic influences account for more than 50% of the variability in adult cognitive abilities[6]. We have shown that genetic variation in some specific genes, e.g. *APOE *is associated with change in cognitive ability with age, but not with the stable trait of intelligence[7]. Therefore, it is likely that some genetic variants are associated with life-long cognitive abilities and others specifically with variance in age-related cognitive decline. The search for genetic contributions to cognitive ageing can be guided by focussing on mechanisms that affect brain ageing[5].

Oxidative stress is hypothesised to be a significant contributor to cellular ageing. The free radical theory of ageing predicts that, with increasing age, free radicals, reactive by-products of oxidative metabolism, damage macromolecules such as DNA, protein and lipids[8,9]. Support for the free radical hypothesis of ageing comes from a wide variety of sources, including analyses of mutations and transgenic animals (for recent reviews see[10,11]). The brain is particularly vulnerable to oxidative damage as a result of its high aerobic metabolism and high concentrations of polyunsaturated fatty acids that are susceptible to lipid peroxidation [12-15].

Oxidative damage to mitochondrial DNA accumulates at a ten-fold higher rate than nuclear DNA, although its precise significance to ageing remains controversial[16,17]. The constant leak of reactive oxygen species from mitochondria increases with age, and deficiency of both mitochondrial and cytoplasmic superoxide dismutase are associated with neurodegeneration due to oxidative damage [18-20]. A role for oxidative stress has been proposed both in Alzheimer's disease (AD), associated with amyloid plaques[21,22], and in Parkinson's disease, with the presence of iron and auto-oxidised monoamines[23]. A role for oxidative stress has also been proposed in mild cognitive impairment[24,25]. Non-pathological cognitive ageing was found to be related to differences in oxidative stress (measured, for example, by thiobarbituric acid reactive substances) in a large community study of older people[26]. It is also implicated in the "common cause hypothesis of ageing": the recent finding that physical and cognitive capabilities are highly correlated in old age[27].

Expression profiling of large gene arrays in adult and aged mouse brain also supports a role for oxidative damage in cognitive ageing[28,29]. Lee et al[28] examined the expression profiles in neocortex and cerebellum of 6,347 genes in adult (5 months) and aged (30 months) mice. In both brain regions, gene expression profiles showed increased inflammatory response and oxidative stress gene expression in the older mice. These authors concluded that oxidative stress is an important and perhaps underlying cause of the ageing process in post-mitotic (neural) tissues. In a similar study, Jiang et al[29] probed over 11,000 genes in cortex and hypothalamus in 2 month and 22 month old mice and found altered expression for 98 genes (0.9%) in cortex, about 20% of which were also altered in hypothalamus. Significant changes (at least two-fold) were found in a variety of proteins, including eight concerned with oxidative stress response.

We previously identified associations between common functional polymorphisms in genes involved in AD or oxidative stress and cognitive ageing[7,30,31]. However, these studies all involved genotyping small numbers of polymorphisms in a small sample of genes. Technology is now available to genotype easily much larger numbers of polymorphisms. The aim of the present study was to investigate the influence of genetic variation in genes primarily related to oxidative stress and antioxidant defences in two cohorts of relatively healthy older individuals. These are the Lothian Birth Cohort of 1921 (LBC1921) and the Aberdeen Birth Cohort of 1936 (ABC1936), on whom cognitive ability test scores are available at age 11 and in later life; that is, they have data on the lifetime trait of intelligence, and lifetime cognitive change[32]. These cohorts form a unique resource to test for genes associated with cognitive ageing. Both cohorts took an identical mental ability test at age 11 and a different but overlapping series of cognitive ability tests at either age 79 (LBC1921) or age 64 (ABC1936)[32]. To utilise this resource a candidate gene genetic association study was performed by genotyping 387 SNPs in 444 members of LBC1921. We have ~80% power to detect an effect size of 3% at a type-1 error rate of 0.01. Replication of possible associations is important. Therefore, SNPs that showed a positive association with either cognitive ability at age 11 or cognitive ageing were then genotyped in 485 members of ABC1936.

## Results

384 SNPs were selected for genotyping by the GoldenGate™ assay. A multiplex assay was successfully designed for 322 SNPs (83.9%). 437 (261 women, 176 men) of the 444 LBC1921 subjects (98.4%) were successfully genotyped for at least 316 SNPs. Genotyping data were obtained, from both samples, for 15 of the 16 subjects who were genotyped in duplicate and no discrepancies were identified. Three further SNPs were genotyped by TaqMan^® ^technology in 424–434 of the subjects. In summary 325 SNPs were genotyped in 420–437 subjects. 86 SNPs (26.5%) were monomorphic in LBC1921.

### LBC1921

#### Childhood cognitive ability in LBC1921

There was a nominally significant association between three SNPs and age 11 Moray House Test (MHT) score: *CTSZ*, rs9760 (F = 5.625, *p *= 0.004, η^2 ^= 0.025); *GSTZ1*, rs3177429 (F = 4.820, *p *= 0.009, η^2 ^= 0.022); *NDUFS4 *rs31304 (F = 9.757, *p *= 0.002, η^2 ^= 0.022). The genotype frequencies for each of these SNPs did not differ significantly from Hardy-Weinberg equilibrium.

#### Cognitive ageing in LBC1921

Table 1 indicates the effect of each polymorphic SNP (*p*-value) on each of the age 79 cognitive outcomes controlling for age 11 MHT score (i.e. the effect on cognitive ageing). Sex was included as a between subjects variable, except in the case of *PRDX4 *SNP rs552105 which is on the X chromosome. For this SNP men and women were analysed separately. Nine SNPs located in eight genes (*APP*, *GLRX*, *HSPA9B*, *MSRB2*, *NDUFS1*, *NDUFV2*, *NDUFV3 *and *NOS1*) showed a nominally significant association (*p *< 0.01) with one of the cognitive variables (table 2). The two SNPs in *NDUFV3 *were in almost complete linkage disequilibrium. Therefore, only rs8128440 was taken forward to the next stage. The minor allele frequency of SNP rs9658446 in *NOS1 *was only 4.58 × 10^-3^, and therefore this SNP was not carried forward to the next stage. The genotype frequencies for each of these SNPs did not differ significantly from Hardy-Weinberg equilibrium.

**Table 1:** Effect of each polymorphic SNP on each of the cognitive outcomes, controlling for sex and age 11 cognitive ability.

		**Moray House Test**	**Raven's Progressive Matrices**	**Verbal Fluency**	**Logical Memory**
**Gene**	**SNP**				

AGER	rs3134943	.770	.808	.058	.773
	rs1800684	.701	.738	.036	.773
APOD	rs6786696	.982	.930	.680	.807
	rs17033096	.748	.683	.527	.610
	rs4686327	.580	.959	.761	.419
APP	rs1787439	.817	.409	.065	.112
	rs2040276	.094	.725	.108	.246
	rs2026225	.736	.818	.770	.142
	rs2830019	.948	.443	.809	.106
	rs2830020	.948	.443	.809	.106
	rs2830038	.284	.636	.076	.075
	rs1041420	.022	.346	.389	.148
	rs2830045	.463	.366	.398	.669
	rs2830048	.247	.789	.233	.052
	rs2830052	.016	.051	.932	.405
	rs3787650	.669	.667	.871	.823
	rs2830071	.474	.903	.469	.119
	rs2830102	**.003**	.016	.978	.436
BACE	rs535860	.994	.777	.416	.610
	rs638405	.541	.184	.985	.359
CAT	rs769217	.304	.804	.121	.921
CBS	rs234706	.149	.299	.846	.676
CDKN1B	rs3093728	.677	.767	.286	.263
	rs34330	.088	.522	.413	.034
	rs4251698	.632	.589	.439	.328
	rs7330	.901	.782	.231	.880
CHRM2	rs8191992 associated with IQ [60].	.156	.610	.796	.781
CP	rs16861582	.020	.228	.728	.318
	rs1053709	.684	.682	.667	.606
	rs6799507	.770	.859	.061	.854
	rs701753	.576	.325	.157	.682
	rs17838831	.155	.103	.786	.027
CRYAB	rs4252581	.376	.683	.243	.789
	rs14133	.931	.546	.696	.552
	rs4252583	.097	.010	.394	.184
	rs762550	.344	.713	.622	.073
CSNK1D	rs6416862	.338	.506	.010	.814
CTSD	rs17571 associated with AD [61] and general intelligence [62].	.355	.402	.958	.333
CTSH	rs13345	.312	.964	.118	.589
	rs12148472	.700	.944	.318	.406
	rs1036938	.835	.509	.131	.916
CTSS	rs10888390	.322	.557	.259	.127
CTSZ	rs9760	.623	.387	.011	.082
DNAJB1	rs3962158	.081	.295	.216	.716
DNAJB2	rs2276638	.283	.639	.794	.321
	rs3731897	.287	.546	.793	.383
FOSB	rs2282695	.661	.938	.317	.762
	rs2238686	.073	.036	.676	.849
FOXO3A	rs12202049	.851	.557	.782	.933
	rs2883881	.831	.952	.250	.098
	rs17532874	.814	.848	.475	.510
	rs12203787	.887	.473	.842	.892
GCLC	rs1555903	.659	.336	.420	.295
GFAP	rs3744473	.620	.275	.362	.674
	rs3744470	.782	.716	.890	.420
	rs9916491	.620	.275	.362	.674
	rs1126642	.669	.187	.295	.321
GLRX	rs4561	.254	.560	.182	**.003**
GPX1	rs3448	.731	.135	.660	.464
GSR	rs2251780	.232	.664	.788	.860
GSS	rs6119545	.019	.029	.073	.299
	rs7265992	.238	.412	.193	.619
	rs2025096	.999	.897	.963	.614
GSTA2	rs6577	.683	.052	.908	.492
	rs2180314	.662	.116	.395	.500
GSTA4	rs1802061	.268	.810	.365	.982
GSTA5	rs2397118	.599	.960	.763	.677
GSTM3	rs7483	.722	.926	.736	.488
GSTM4	rs560018	.747	.619	.842	.773
	rs650985	.763	.756	.686	.717
GSTO1	s4925	.344	.971	.847	.119
GSTO2	rs156697	.619	.726	.405	.028
	rs3758572	.670	.553	.407	.754
GSTP1	rs762803	.258	.969	.712	.688
	rs947894	.206	.302	.745	.395
	rs1799811	.436	.528	.110	.171
	rs1871042	.269	.500	.987	.461
GSTT2	rs140188	.266	.382	.070	.297
GSTZ1	rs2270421	.072	.203	.178	.847
	rs2287395	.159	.288	.174	.667
	rs3177429	.245	.507	.533	.737
	rs2287396	.267	.083	.884	.268
	rs1046428	.312	.511	.191	.663
HMOX2	rs6500610	.635	.364	.275	.215
	rs11643057	.733	.411	.666	.363
	rs17137094	.010	.011	.189	.675
HSPA12A	rs1665659	.443	.454	.067	.362
	rs4752003	.030	.010	.619	.689
	rs1665638	.783	.645	.270	.293
	rs740599	.585	.630	.865	.758
	rs1900501	.247	.025	.435	.190
HSPA12B	rs3827077	.121	.689	.473	.190
	rs6076550	.493	.505	.828	.870
	rs2295340	.516	.725	.462	.612
HSPA1L	rs2075800	.133	.371	.267	.845
HSPA2	rs17101915	.493	.583	.051	.249
	rs11848114	.251	.615	.109	.110
HSPA4	rs398606	.680	.021	.216	.775
	rs14355	.096	.062	.716	.246
HSPA5	rs430397	.348	.413	.084	.825
HSPA8	rs3763897	.349	.020	.119	.960
HSPA9B	rs10117	**.006**	.211	.261	.267
HTR2A	rs3803189	.128	.118	.984	.910
	rs6314 associated with episodic memory [63].	.096	.180	.429	.388
	rs1923884	.966	.601	.948	.773
	rs6305	.151	.746	.926	.133
	rs6313 associated with AD [64].	.209	.079	.996	.885
IDE	rs7895832	.181	.093	.690	.470
	rs3758505 associated with AD [65].	.181	.093	.690	.470
IL1B	rs1143634 associated with AD [66].	.372	.082	.738	.450
	rs16062	.508	.404	.711	.484
	rs1143627	.591	.872	.354	.926
LTF	rs4683233	.220	.073	.826	.023
MPO	rs2759	.500	.154	.634	.301
	rs7208693	.959	.940	.782	.945
MSRA	rs12679328	.950	.072	.466	.063
	rs3735823	.985	.087	.833	.245
	rs814422	.237	.111	.462	.584
	rs1994224	.460	.078	.592	.414
	rs6601414	.034	.386	.211	.717
	rs17151140	.396	.191	.876	.567
	rs1484645	.609	.343	.252	.039
	rs6986977	.510	.907	.764	.261
	rs877390	.661	.389	.544	.690
	rs7845503	.437	.936	.722	.020
	rs6992349	.956	.718	.292	.573
	rs4288376	.189	.373	.503	.250
	rs10503405	.965	.353	.263	.871
	rs6983870	.271	.246	.432	.265
	rs4260895	.263	.069	.341	.154
	rs2952182	.355	.612	.586	.832
	rs11783821	.437	.523	.149	.586
	rs17151588	.204	.360	.309	.214
	rs7832708	.233	.151	.899	.021
	rs4841322	.746	.663	.882	.400
	rs4841324	.706	.644	.849	.435
MSRB2	rs10764383	.951	.540	.043	.272
	rs11013295	.862	.668	.404	.354
	rs7427	**.006**	.111	.487	.550
NDRG1	rs2977499	.536	.626	.436	.829
	rs2272653	.970	.517	.812	.184
	rs2930002	.961	.599	.502	.543
NDUFA10	rs2083411	.594	.085	.809	.255
NDUFA3	rs254259	.021	.020	.517	.269
NDUFA6	rs1801311	.630	.207	.074	.036
NDUFA7	rs561	.417	.734	.077	.754
	rs2241591	.239	.180	.366	.774
NDUFA8	rs4147659	.180	.585	.584	.574
	rs6822	.238	.634	.690	.646
	rs4679	.079	.592	.405	.389
NDUFA9	rs4147672	.611	.387	.825	.723
	rs4147682	.611	.387	.825	.723
NDUFAB1	rs459894	.620	.580	.948	.070
NDUFAF1	rs3204853	.294	.162	.506	.566
NDUFB10	rs2302175	.129	.533	.878	.373
NDUFB5	rs2339844	.590	.320	.894	.083
NDUFB7	rs9543	.676	.552	.081	.032
NDUFB8	rs1800662	.354	.447	.709	.738
NDUFB9	rs11547284	.483	.023	.690	.840
NDUFS1	rs11548670	.166	.258	**.002**	.287
	rs4147707	.977	.610	.613	.993
NDUFS2	rs3813624	.225	.973	.255	.783
	rs16832694	.490	.890	.878	.229
	rs16832699	.225	.973	.255	.783
	rs11587213	.957	.200	.293	.925
NDUFS4	rs4147732	.727	.369	.422	.516
	rs2279516	.710	.876	.073	.240
	rs13156337	.417	.608	.468	.075
	rs31304	.451	.409	.938	.341
	rs31303	.783	.260	.609	.885
	rs567	.688	.190	.226	.641
NDUFS6	rs3776141	.329	.561	.117	.011
NDUFV2	rs906807	.346	**.009**	.892	.732
NDUFV3	rs4148973	.718	.473	**.0003**	.559
	rs8128440	.710	.500	**.0002**	.742
NOS1	rs9658501	.278	.275	.696	.799
	rs3741475	.556	.447	.469	.212
	rs10774909	.393	.138	.533	.338
	rs9658446	.062	.186	.249	**.004**
	rs2293054	.443	.289	.893	.718
	rs11612772	.659	.255	.253	.737
	rs561712	.795	.719	.870	.628
	rs9658256	.661	.270	.405	.089
NOS2A	rs2297512	.187	.471	.553	.164
	rs2297518	.504	.285	.556	.254
	rs1137933	.455	.429	.206	.428
	rs3730017	.318	.955	.342	.373
NOS3	rs1549758	.179	.489	.620	.463
	rs1799983 associated with mild cognitive impairment [67].	.380	.263	.779	.258
	rs2566514	.738	.774	.876	.298
	rs3918232	.612	.092	.226	.546
NR2C2	rs17536979	.480	.367	.719	.206
	rs648912	.957	.489	.849	.358
PLAU	rs2227564 associated with AD [68].	.766	.877	.796	.623
	rs2227567	.121	.816	.508	.440
	rs2227568	.974	.696	.053	.886
	rs4065	.459	.953	.120	.293
PON2	rs6954345	.054	.261	.510	.788
	rs10487133	.294	.661	.510	.686
	rs11545941	.054	.261	.510	.788
	rs17166875	.054	.261	.510	.788
PRDX1	rs6667191	.912	.697	.763	.689
PRDX2	rs10413408	.824	.251	.445	.773
	rs10422248	.824	.251	.445	.773
PRDX4*	rs552105 (male)	.611	.942	.495	.509
	rs552105 (female)	.856	.569	.894	.898
	rs1548734 (male)	.611	.942	.495	.509
	rs1548734 (female)	.832	.515	.891	.870
SAA2	rs2468844	.558	.557	.676	.596
SEPP1	rs6413428	.073	.055	.948	.307
	rs7579	.616	.677	.851	.786
SIRT1	rs2273773	.967	.517	.358	.730
	rs2234975	.937	.840	.454	.750
SLC25A27	rs9369628	.383	.213	.990	.646
	rs12192544	.881	.251	.989	.739
	rs3757241	.126	.975	.304	.739
SOD2	rs1799725	.381	.937	.438	.111
SOD3	rs1799895	.485	.813	.745	.924
TF	rs1130459	.153	.069	.260	.311
	rs1799852	.685	.237	.149	.919
	rs1799899	.789	.722	.454	.835
	rs1049296	.434	.899	.829	.621
	rs3811656	.087	.025	.248	.796
TXN	rs4135162	.157	.747	.929	.289
TXN2	rs2281082	.951	.220	.864	.253
TXNRD1	rs11111979	.755	.026	.399	.709
	rs7134193	.850	.101	.314	.737
	rs4964287	.924	.205	.035	.615
TXNRD2	rs3827288	.585	.211	.136	.471
	rs5992495	.879	.865	.209	.056
	rs5748469	.577	.780	.232	.832
	rs5746847	.388	.983	.125	.691
TXNRD3	rs777241	.993	.767	.270	.498
UCP2	rs660339	.578	.723	.520	.372
VEGF	rs2010963	.980	.766	.372	.228
	rs833068	.974	.765	.434	.205
	rs3025000	.849	.828	.408	.227
	rs3025010	.192	.935	.267	.161
	rs3025039	.112	.130	.882	.230
	rs3025053	.487	.326	.705	.620
VIM	rs1049341	.336	.976	.815	.887

*p *values are given. *p *values < 0.01 are in bold. SNPs previously associated with intelligence or AD are indicated.

*PRDX4 is located on the X chromosome and therefore men and women were analyzed separately.

**Table 2:** SNPs showing a significant (p < 0.01) association with at least one cognitive trait at age 79 (LBC1921), controlling for sex and age 11 cognitive ability.

		**No. of subjects with each genotype**			**Moray House Test**			**Raven's Progressive Matrices**			**Verbal Fluency**			**Logical Memory**		
**Gene**	**SNP**	**A/A**	**A/B**	**B/B**	F	*p*	η^2^	F	*p*	η^2^	F	*p*	η^2^	F	*p*	η^2^

APP	rs2830102	46	177	214	5.835	**.003**	.026	4.163	.016	.019	.023	.978	.000	.831	.436	.004
GLRX	rs4561	160	222	54	1.375	.254	.006	.580	.560	.003	1.712	.182	.008	5.893	**.003**	.027
HSPA9B	rs10117	66	217	154	5.194	**.006**	.024	1.563	.211	.007	1.349	.261	.006	1.326	.267	.006
MSRB2	rs7427	53	200	184	5.099	**.006**	.023	2.212	.111	.010	.722	.487	.003	.598	.550	.003
NDUFS1	rs11548670	412	25	0	1.922	.166	.004	1.281	.258	.003	9.629	**.002**	.022	1.135	.287	.003
NDUFV2	rs906807	17	134	286	1.063	.346	.005	4.733	**.009**	.022	.114	.892	.001	.312	.732	.001
NDUFV3	rs4148973	54	196	187	.332	.718	.002	.749	.473	.003	8.379	**.0003**	.038	.583	.559	.003
NDUFV3	rs8128440	186	195	55	.342	.710	.002	.694	.500	.003	8.816	**.0002**	.039	.299	.742	.001
NOS1	rs9658446	0	4	433	3.491	.062	.008	1.754	.186	.004	1.334	.249	.003	8.567	**.004**	.019

*p *values < 0.01 are highlighted in bold.

#### Cognitive ability and ageing in ABC1936

Nine of the 10 SNPs that showed a positive association in LBC1921 with either age 11 cognitive ability or cognitive ageing were successfully genotyped in ABC1936 by KBiosciences. The *APP *SNP rs2830102 was genotyped using TaqMan^® ^technology. None of the SNPs were significantly associated with either age 11 MHT score or cognitive ageing in ABC1936 (*p *> 0.01). Table 3 shows the effect of SNPs showing a positive association with at least one cognitive trait at age 79 (LBC1921), controlling for sex and age 11 cognitive ability, on cognitive traits at age 64 (ABC1936), controlling for sex and age 11 cognitive ability. The genotype frequencies for each of these SNPs did not differ significantly from Hardy-Weinberg equilibrium.

**Table 3:** Effect of SNPs showing a significant (*p *< 0.01) association with at least one cognitive trait at age 79 (LBC1921), controlling for sex and age 11 cognitive ability, on cognitive traits at age 64 (ABC1936), controlling for sex and age 11 cognitive ability.

		**No. of subjects with each genotype**			**BD**	**DS**	**RM**	**UCO**	**AVLT**
**Gene**	**SNP**	**A/A**	**A/B**	**B/B**					

APP	rs2830102	29	168	167	.380	.855	.345	.167	.984
GLRX	rs4561	128	171	66	.465	.426	.391	.044	.848
HSPA9B	rs10117	56	180	142	.433	.963	.012	.841	.698
MSRB2	rs7427	41	164	174	.451	.585	.813	.484	.650
NDUFS1	rs11548670	351	25	1	.570	.113	.956	.395	.144
NDUFV2	rs906807	11	102	256	.953	.749	.783	.410	.672
NDUFV3	rs8128440	145	178	41	.856	.903	.509	.146	.515

*p *values are given.

Key: BD = Block Design, DS = Digit Symbol, RM = Raven's Progressive Matrices, UCO = Use of Common Objects, AVLT = Auditory Verbal Learning Test

#### A combined LBC1921/ABC1936 analysis to detect associations with cognitive ageing

**Figure 1:**
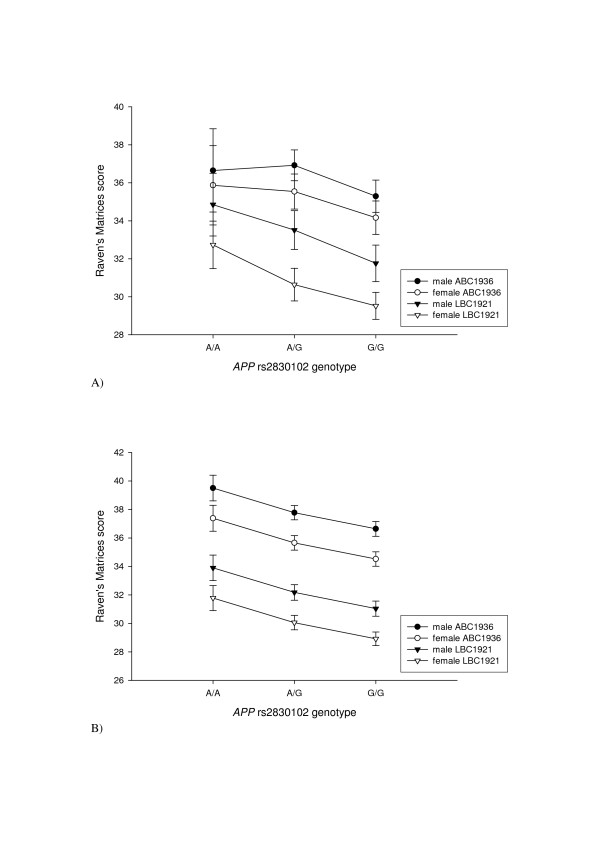
Score on Raven's Matrices by *APP *rs2830102 genotype, sex and cohort (LBC1921 or ABC1936): A) raw data; B) estimated marginal means from general linear model, adjusted for age 11 MHT score.

Because larger sample sizes have greater power to detect associations, general linear modelling was performed using combined data from LBC1921 and ABC1936 to investigate the effect of the seven SNPs that showed a significant association with cognitive ageing in LBC1921, on a relatively large sample size (n = 858–886). An effect size of just 2% can be detected with > 80% power at a type-1 error rate of 0.01 using 858 subjects. The effect size of any single polymorphism influencing variation in a complex trait like cognitive ageing may well be relatively small, as many polymorphisms are likely to be involved[5]. A combined LBC1921/ABC1936 univariate analysis was performed for the each of these seven SNP genotypes, with later life Raven's Progressive Matrices score (the only later life cognitive test that was measured in both cohorts) as the dependent variable. All the cognitive tests used to assess LBC1921 are significantly positively correlated[33] and, therefore, associations that were previously identified with tests other than Raven score may be detected with this test when using a larger sample size. Other effects included in the model were age 11 MHT score, sex and cohort (table 4). All interactions were non-significant and removed from the models. As previously shown[31] cohort and sex were significant for all SNP models (*p *< 0.001), with ABC1936 and males scoring higher than LBC1921 and females. Age 11 MHT score contributed significantly to later life Raven score (*p *< 0.001). This latter finding reflects the highly significant partial correlation between age 11 MHT score and later life Raven score, controlling for cohort (r = 0.52, df = 892, *p *< 0.001). *APP *intronic SNP, rs2830102, was significantly associated with later life Raven score, controlling for age 11 MHT score, sex and cohort (F = 5.988, *p *= 0.003, η^2 ^= 0.014). Figure 1 shows the Raven score raw data (A), and the estimated marginal means (B), for later life Raven scores by sex and cohort, controlling for age 11 MHT score. G/G (genotype B/B in tables 2, 3 and 4) homozygotes scored significantly lower than both heterozygotes (*p *= 0.029) and A/A (genotype A/A in tables 2, 3 and 4) homozygotes (*p *= 0.002). There was a trend for heterozygotes to score lower than A/A homozygotes (*p *= 0.057). None of the other SNP genotypes were significantly associated with later life Raven score, controlling for age 11 MHT, sex and cohort (*p *> 0.01).

**Table 4:** Effect of SNPs showing a significant (*p *< 0.01) association with at least one cognitive trait at age 79 (LBC1921), controlling for sex and age 11 cognitive ability, on Raven's Progressive Matrices Score in later life controlling for sex, age 11 cognitive ability and cohort (ABC1936 or LBC1921).

		**No. of subjects with each genotype**			**Raven's Progressive Matrices**		
**Gene**	**SNP**	**A/A**	**A/B**	**B/B**	F	*p*	η^2^

APP	rs2830102	79	381	415	5.988	**.003**	.014
GLRX	rs4561	323	424	127	.846	.429	.002
HSPA9B	rs10117	133	433	323	1.528	.217	.003
MSRB2	rs7427	106	396	386	1.443	.237	.003
NDUFS1	rs11548670	829	56	1	.533	.587	.001
NDUFV2	rs906807	30	252	595	3.625	.027	.008
NDUFV3	rs8128440	353	414	104	.736	.479	.002

*p *values < 0.01 are highlighted in bold.

## Discussion

To our knowledge, this is the first large-scale investigation into the possible genetic contributions to the normal variability in cognitive ageing experienced by individuals. We examined genes previously implicated in oxidative stress, dementia and cognitive function. Of 325 gene variants analysed, nine were positively associated with variation in performance on one of four tests of cognitive ability at age 79 (LBC1921), controlling for sex and childhood cognitive ability. Two of these SNPs were in strong linkage disequilibrium and one SNP had a very low minor allele frequency. None of these associations was replicated in a second cohort of 64 year olds (ABC1936) who took a different but overlapping series of cognitive tests. Therefore, the present study should be considered as an informative, null study concerning a coherent set of genes that might have, but do not, affect normal cognitive ageing, beyond the effect size which it was powered to detect.

However, *APP *intronic SNP rs2830102 genotype was associated with non-verbal reasoning, as measured by Raven's Progressive Matrices, in a joint analysis of LBC1921 and ABC1936 data. It is emphasised, though, that it did not have significant effects in both cohorts separately, and as such it needs replicating in other cohorts. *APP *genotype accounted for 1.4% of the variance in the Raven scores, after adjustment for sex, cohort and childhood ability differences. Although this is a relatively small effect size, it is what is expected with a complex trait like cognitive ageing, where many variants are likely to be involved. *APOE*, which is one of the few genes that has been associated with cognitive ageing in several cohorts, including LBC1921[7], has an effect size of just 1% to 2% and is considered to be important. 12.4% of the variance was accounted for by the cohort of the participants. ABC1936 participants who, at age 64, were 15 years younger, scored significantly better than the 79-year-olds in the LBC1921 (*p *< 0.001). No significant interaction between year of cohort and *APP *genotype was identified.

*APP *encodes the amyloid β (Aβ) precursor protein. Extracellular Aβ plaques, which form in the meningeal vessels of AD patient brains, are a defining feature of the disease. Mutations in both the coding region[34] and the promoter region[35] of this gene have been associated with AD. Aberrant expression of APP has also been implicated in AD[36,37]. AD is characterised by an impairment of multiple cognitive domains. Amyloidogenic peptide derivatives of mutant APP have been implicated in the generation of free radicals and with mitochondrial oxidative damage (reviewed in[38]). It is possible that common variation in *APP *DNA sequence is associated with variation in oxidative stress in the general population, leading to variation in normal cognitive ageing. This may reflect the possibility that the neurobiology of both cognitive ageing and AD is, to some extent, a continuum. It is also possible that the association between *APP *SNP rs2830102 and normal cognitive ageing, as measured using Raven's Progressive Matrices, is related to incipient AD in some members of LBC1921 and ABC1936.

SNP rs2830102 is located in intron 1 of *APP *and may affect regulation of gene expression. Although the SNP does not lie in a predicted promoter region[39] and is not predicted to alter splicing it does occur within a region of sequence conservation[40]. Alternatively it may be in linkage disequilibrium with another functional SNP, possibly in the promoter of the gene. It is important that the *APP *gene is investigated further for its role in both non-pathological cognitive ageing and AD.

Several of the SNPs investigated in this study had previously been associated with intelligence or AD (table 1). We failed to find any significant association between these SNPs and either cognitive ability at age 11 or cognitive ageing in LBC1921. Such attempted replications are important, because initial reports of genotype-phenotype associations often do not replicate.

To investigate genetic influences on non-pathological cognitive ageing we chose to perform a relatively large scale genetic association study using candidate genes, for which there was strong a priori evidence for their involvement in brain ageing. We focussed on a specific ageing-related mechanism, that of oxidative stress. We were in the invaluable position of being able to test directly for cognitive ageing across a long period of time, as we had cognitive ability scores at both age 11 and in later life. There has been much discussion in the literature regarding larger scale association study designs. We chose a candidate genes approach that allowed the use of smaller numbers of SNPs compared to a whole genome association study. However, it is likely that important regions of the genome were missed by this approach. We followed recent guidelines from a genomewide association scan workshop[41] that concluded that multistage designs, whereby a sub-set of subjects are initially genotyped and additional subjects are then genotyped for SNPs that show a positive association, enhanced the efficiency of such studies. We chose to genotype a limited number of potentially functional SNPs in a larger number of genes rather than to attempt to fully cover a smaller number of genes using, for example, tagging SNPs and may therefore have missed important SNPs whose functionality was not predicted. We considered this a more efficient use of limited genotyping funds. It allowed us to cover more of our candidate genes and increased the likelihood that we would identify a causative SNP, particularly as concern exists over the portability of tagging SNPs across populations. A few recent preliminary studies indicate that it may be possible to use tagging SNPs designed in one population to investigate associations in a second population, but this should only be done with caution [42-45]. With regard to the analysis, we decided to initially concentrate on the identification of individual SNPs that have a detectable main effect on variation in cognitive ageing. However, in the future we may include newly developed statistical techniques that allow the identification of interlocus interactions[46].

Like all large scale genetic association studies, this study suffers from the problem of multiple testing; we initially investigated 325 SNPs and four cognitive tests in 437 subjects. Because many of the SNPs are in linkage disequilibrium and, moreover, scores on the cognitive tests are positively correlated, it was deemed inappropriate to perform a Bonferroni-type correction. However, we were able to genotype SNPs showing a nominally significant association in the first cohort, with a second equally large and valuable cohort and, we used a relatively stringent *p *valueof < 0.01.

It is also important, given the relatively small size and younger age of the replication cohort (n = 485), that SNPs that showed a positive association in LBC1921, but not in ABC1936 or the combined cohorts, are investigated in future association studies to identify genetic determinants of cognitive ageing. A further caveat of the study is that ABC1936 did not take exactly the same cognitive tests as LBC1921. Therefore, associations identified in LBC1921 may have been with specific cognitive abilities that were not examined in ABC1936.

## Conclusion

This study has identified a number of genes, for which there was strong a priori evidence for their involvement in cognitive ageing, which have an association with cognitive ageing in a cohort of relatively healthy 79 year old subjects (LBC1921). A significant association with a SNP in the gene encoding APP was also identified in a combined analysis of LBC1921 and a second younger cohort (ABC1936), suggesting its importance in cognitive ageing as well as AD. It is important that the role of this gene in cognitive ageing is investigated further.

## Methods

### Subjects

The subjects recruited to this study originally participated, at the age of about 11 years, in the Scottish Mental Surveys of either 1932 or 1947[32,47,48]. On June 1^st ^1932 and June 4^th ^1947 a valid mental ability test, a version of the Moray House Test No. 12 (MHT), was given to almost all Scottish children attending school on the Survey day who were born in 1921 (N = 87,498) or 1936 (N = 70,805), respectively.

#### Lothian Birth Cohort 1921 (LBC1921)

LBC1921 are surviving participants of the Scottish Mental Survey of 1932, who were living independently in the Edinburgh area at the time of recruitment. Further testing and recruitment details have been published previously[32]. Mean age at re-test was 79.1 years (SD = 0.6 years), and all subjects were Caucasian. The following inclusion criteria were applied: Cognitive ability scores were available at age 11 and age 79; there was no history of dementia; Mini-Mental State Examination (MMSE) score was 24 or greater; and SNP genotyping was successful. This gave a total of 437 subjects (261 women, 176 men).

#### Aberdeen Birth Cohort 1936 (ABC1936)

ABC1936 are surviving participants of the Scottish Mental Survey of 1947, who were living independently in the city of Aberdeen at the time of recruitment. Further recruitment details have been published previously[49,50]. Mean age at re-test was 64.6 years (SD = 0.7 years), and all subjects were Caucasian. The following inclusion criteria were applied: Cognitive ability scores were available at age 11 and age 64 and Mini-Mental State Examination (MMSE) score was 24 or greater. This gave a total of 485 subjects (246 women, 239 men).

### Cognitive testing

#### Moray House Test No. 1(MHT)

All subjects took this general mental ability test at age 11, in the Scottish Mental Surveys of 1932 and 1947. LBC1921 re-took the test at about age 79. The test is described fully elsewhere[1,32,47]. The same instructions and the time limit (45 minutes) were used on both occasions. At re-test, ABC1936 took subtests of the Wechsler Adult Intelligence Scale-Revised instead of the MHT[51]: the Block Design, which measures visuo-spatial ability, and Digit Symbol, which measures speed of information processing[51].

#### Mini-Mental State Examination (MMSE)

MMSE[52] was used to screen both cohorts for possible dementia. Maximum score is 30. A score of less than 24 was used here as an exclusion criterion because it is often adopted as an indicator of possible dementia.

Both cohorts underwent a series of mental tests designed to examine different cognitive functions: non-verbal reasoning, executive function, and memory and learning. We have previously described this testing in detail[32,53]. The individual cognitive functions of the two independent cohorts (LBC1921 and ABC1936) were examined using a different series of tests as indicated below:

#### Non-verbal reasoning

##### Raven's Progressive Matrices[54]

Non-verbal reasoning was examined in all subjects using Raven's Standard Progressive Matrices. The time limit was 20 minutes.

#### Executive Function

##### Verbal fluency

LBC1921 took the verbal fluency test, which is described as a test of prefrontal executive function[55,56].

##### Uses of Common Objects

ABC1936 took the use of common objects test, which is described as a test of executive function or purposive action[55].

#### Verbal Memory and Learning

##### Logical Memory

LBC1921 took the Logical Memory test, which is a verbal declarative memory sub-test from Wechsler Memory Scale-Revised[57].

##### Rey Auditory Verbal Learning Test

ABC1936 took the auditory verbal learning test which assesses short and longer term memory and learning[55].

#### Illumina SNP selection

A list of 141 brain-expressed genes was selected and provided to Illumina (table 5). They were selected if they were: a) implicated in antioxidant defence; b) vitagenes (longevity assurance processes); c) associated with cognitive function; d) associated with AD; e) "stress response" genes showing an increased expression in the aged mouse [28]; and/or f) nuclear genes encoding mitochondrial complex 1 proteins. From an initial list of 14,033 potential SNPs, 384 were selected for genotyping using the following criteria: a) all designable (including designability score 0.5) SNPs previously associated with AD and cognitive function; b) all designable (including designability score 0.5) functional SNPs; c) all non-synonymous validated and designable (including designability score 0.5) SNPs; d) all validated and designable (including designability score 0.5) SNPs at exon/intron boundaries that potentially alter splicing; e) all validated and designable (including designability score 0.5) SNPs with percentage identity in mouse >= 80%; f) all validated and designable (excluding designability score 0.5) SNPs with percentage identity in mouse between 60% and 80%; g) remaining SNPs were Illumina validated synonymous SNPs in previously unrepresented genes (see additional file 1). Designability is ranked as 0, 0.5 or 1. A "0" is assigned to SNPs for which an assay cannot be designed, "0.5" indicates the SNP has a designability score low enough to suggest that there might be challenges to the design, and "1" is reserved for those that do not appear to have any challenges in their designability. Validation class is ranked as 1, 2, or 3. "1" means that a SNP is nonvalidated, "2" is a two-hit SNP (non Illumina validated, i.e. it has been validated on some other platform on more than one chromosome), and "3" means two-hit Illumina validated. The percentage identity with mouse is based on a 120 base pair window surrounding the SNP.

**Table 5:** Cognitive Ageing Candidate Genes (expressed in the brain).

**gene symbol**	**gene name and function**
**antioxidant defence genes**	

BACE1	beta-site APP-cleaving enzyme 1. Responsible for the proteolytic processing of the amyloid precursor protein (APP).
CAT	catalase. Protects cells from the toxic effects of hydrogen peroxide. Contains functional promoter polymorphism [69].
CBS	cystathionine-beta-synthase.
CCS	copper chaperone for SOD. Delivers Cu/Zn to SOD1
CDKN1B	cyclin-dependent kinase inhibitor 1B (p27, Kip1). Involved in G1 arrest.
CP	ceruloplasmin. Ceruloplasmin is a blue, copper-binding (6–7 atoms per molecule) glycoprotein found in plasma. Four possible functions are ferroxidase activity, amine oxidase activity, copper transport and homeostasis, and superoxide dismutase activity.
FOXO3A	forkhead transcription factor (homologue of C elegans daf-16). May trigger apoptosis.
FTH1	ferritin, heavy polypeptide 1. Ferritin is an intracellular molecule that stores iron in a soluble, nontoxic, readily available form.
FTL	ferritin light polypeptide.
FXN	frataxin. Defects in FXN are the cause of Friedreich's ataxia. Probably involved in iron homeostasis.
GCLC	glutamate-cysteine ligase, catalytic subunit. The first rate-limiting enzyme in glutathione biosynthesis.
GGT1	gamma-glutamyltransferase 1. Initiates extracellular gluthatione (GSH) breakdown, provides cells with a local cysteine supply and contributes to maintain intracelular GSH level.
GLRX	glutaredoxin (thioltransferase). GLRX has a glutathione-disulfide oxidoreductase activity in the presence of NADPH and glutathione reductase. Reduces low molecular weight disulfides and proteins.
GLRX2	glutaredoxin 2 (mitochondrial). Catalyses the reversible oxidation and glutathionylation of mitochondrial membrane thiol proteins. Implicated in the protection of mitochondria from ROS.
GPX1	glutathione peroxidase 1 (cytosolic). GPX catalyzes the reduction of hydrogen peroxide, organic hydroperoxide, and lipid peroxides by reduced glutathione and functions in the protection of cells against oxidative damage. Selinium in the form of selenocysteine is part of its catalytic site. GPX1 protects the hemoglobin in erythrocytes from oxidative breakdown. Can be targetted to mitochondria
GPX3	glutathione peroxidase 3 (plasma).
GPX4	glutathione peroxidase 4 (membrane associated phospholipid hydroperoxide GPX). Could play a major role in protecting mammals from the toxicity of ingested lipid hydroperoxides. Essential for embryonic development. Can be targetted to the mitochondria.
GSR	glutathione reductase. Maintains high levels of reduced glutathione in the cytosol.
GSS	glutathione synthetase. The second rate-limiting enzyme in glutathione biosynthesis.
GSTA1	glutathione S-transferase A1. GSTs are a family of phase II enzymes that utilize glutathione in reactions contributing to the transformation of a wide range of exogenous and endogenous compounds, including carcinogens, therapeutic drugs, and products of oxidative stress.
GSTA2	glutathione S-transferase A2.
GSTA3	glutathione S-transferase A3.
GSTA4	glutathione S-transferase A4.
GSTA5	glutathione S-transferase A5.
GSTK1	glutathione S-transferase kappa 1.
GSTM1	glutathione S-transferase M1.
GSTM3	glutathione S-transferase M3 (brain).
GSTM4	glutathione S-transferase M4.
GSTM5	glutathione S-transferase M5.
GSTO1	glutathione S-transferase omega 1. GSTO1 exhibits glutathione-dependent thiol transferase and dehydroascorbate reductase activities. May have a significant housekeeping function, such as protection from oxidative stress.
GSTO2	glutathione S-transferase omega 2.
GSTP1	glutathione S-transferase pi.
GSTT1	glutathione S-transferase theta 1.
GSTT2	glutathione S-transferase theta 2.
GSTZ1	glutathione transferase zeta 1 (maleylacetoacetate isomerase).
LTF	lactotransferrin.
MPO	myeloperoxidase. Part of the host defence system of polymorphonuclear leukocytes. It is responsible for microbicidal activity against a wide range of organisms. In the stimulated PMN, MPO catalyzes the production of hypohalous acids, primarily hypochlorous acid in physiologic situations, and other toxic intermediates that greatly enhance PMN microbicidal activity.
MSRA	methionine sulfoxide reductase A. Has an important function as a repair enzyme for proteins that have been inactivated by oxidation. Catalyzes the reversible oxidation-reduction of methionine sulfoxide in proteins to methionine.
MSRB	methionine sulfoxide reductase B.
NOS1	nitric oxide synthase 1 (neuronal) (mtNOS). Produces nitric oxide (NO) a free radical messenger molecule. NO regulates mitochondrial respiration.
NOS2A	nitric oxide synthase 2A (inducible, hepatocytes).
NOS2B	nitric oxide synthase 2B.
NOS2C	nitric oxide synthase 2C.
NOS3	nitric oxide synthase 3 (endothelial cell). Polymorphism associated with mild cognitive impairment [67].
PON2	paraoxonase 2. Hydrolyzes the toxic metabolites of a variety of organophosphorus insecticides. Capable of hydrolyzing a broad spectrum of organophosphate substrates and a number of aromatic carboxylic acid esters (By similarity). Has antioxidant activity. Is not associated with high density lipoprotein. Prevents LDL lipid peroxidation, reverses the oxidation of mildly oxidized LDL, and inhibits the ability of MM-LDL to induce monocyte chemotaxis.
PRDX1	peroxiredoxin 1. PRDX (a thioredoxin peroxidase) reduces hydrogen peroxide and alkyl hydroperoxide to water and alcohol respectively. Involved in redox regulation of the cell. Reduces peroxides with reducing equivalents provided through the thioredoxin system but not from glutaredoxin. May play an important role in eliminating peroxides generated during metabolism. Might participate in the signaling cascades of growth factors and tumor necrosis factor-alpha by regulating the intracellular concentrations of H(2)O(2).
PRDX2	peroxiredoxin 2.
PRDX3	peroxiredoxin 3 (mitochondrial).
PRDX4	peroxiredoxin 4.
PRDX5	peroxiredoxin 5 (mitochondrial, peroxisomal and cytoplasmic).
PRDX6	peroxiredoxin 6. PRDX6 mutant mice are susceptible to oxidative stress.
SEPP1	selenoprotein P, plasma, 1. Might be responsible for some of the extracellular antioxidant defence properties of selenium or might be involved in the transport of selenium. May supply selenium to tissues such as brain and testis.
SIRT1	sirtuin (silent mating type information regulation 2 homolog) 1 (S. cerevisiae) controls the cellular response to stress by regulating the FOXO family. SIRT1 and FOXO3 form a complex in cells in response to oxidative stress.
SLC25A27	solute carrier family 25, member 27. (UCP4)
SOD1	superoxide dismutase 1 (cytoplasmic). SOD catalyses the formation of hydrogen peroxide and oxygen from superoxide, and thus protects against superoxide-induced damage.
SOD2	superoxide dismutase 2 (mitochondria)
SOD3	superoxide dismutase 3 (extracellular)
TF	transferrin. Transferrins are iron binding transport proteins which can bind two atoms of ferric iron in association with the binding of an anion, usually bicarbonate. It is responsible for the transport of iron from sites of absorption and heme degradation to those of storage and utilization. Serum transferrin may also have a further role in stimulating cell proliferation.
TXN	thioredoxin. Participates in various redox reactions through the reversible oxidation of its active center dithiol to a disulfide and catalyzes dithiol-disulfide exchange reactions.
TXN2	thioredoxin 2 (mitochondrial). A mitochondrial protein-disulphide oxidoreductase essential for control of cell survival during mammalian embryonic development.
TXNRD1	thioredoxin reductase 1.
TXNRD2	thioredoxin reductase 2 (mitochondrial). Maintains thioredoxin in a reduced state. Implicated in the defences against oxidative stress.
TXNRD3	thioredoxin reductase 3.
UCP2	uncoupling protein 2 (mitochondrial, proton carrier). UCP are mitochondrial transporter proteins that create proton leaks across the inner mitochondrial membrane, thus uncoupling oxidative phosphorylation from ATP synthesis. As a result, energy is dissipated in the form of heat.
	
**Vitagenes (longevity assurance processes-chaperones)**	
HMOX1	heme oxygenase (decycling) 1(HSP32) (stress induced). Heme oxygenase cleaves the heme ring at the alpha methene bridge to form biliverdin. Biliverdin is subsequently converted to bilirubin (an antioxidant) by biliverdin reductase.
HMOX2	heme oxygenase (decycling) 2 (constitutive).
HSPA1A	heat shock 70 kDa protein 1A. Member of the HSP70 family. HSP70s stabilize preexistent proteins against aggregation and mediate the folding of newly translated polypeptides in the cytosol as well as within organelles. The HSP70s in mitochondria and the endoplasmic reticulum play an additional role by providing a driving force for protein translocation. They are involved in signal transduction pathways in cooperation with HSP90. They participate in all these processes through their ability to recognize nonnative conformations of other proteins. They bind extended peptide segments with a net hydrophobic character exposed by polypeptides during translation and membrane translocation, or following stress-induced damage.
HSPA1B	heat shock 70 kDa protein 1B.
HSPA1L	heat shock 70 kDa protein 1-like.
HSPA2	heat shock 70 kDa protein 2.
HSPA4	heat shock 70 kDa protein 4.
HSPA5	heat shock 70 kDa protein 5 (glucose-regulated protein, 78 kDa).
HSPA6	heat shock 70 kDa protein 6 (HSP70B').
HSPA8	heat shock 70 kDa protein 8. Polymorphism associated with mild mental impairement [70].
HSPA9B	heat shock 70 kDa protein 9B (mortalin-2). Implicated in the control of cell proliferation and cellular aging. May also act as a chaperone.
HSPA12A	heat shock 70 kDa protein 12A.
HSPA12B	heat shock 70 kD protein 12B.
HSPA14	heat shock 70 kDa protein 14.
	
**genes associated with cognitive function**	
AR	androgen receptor. The steroid hormones and their receptors are involved in the regulation of eukaryotic gene expression and affect cellular proliferation and differentiation in target tissues. CAG repeat polymorphism is associated with cognitive function in older men [71].
CHRM2	cholinergic muscarinic 2 receptor. The muscarinic acetylcholine receptor mediates various cellular responses, including inhibition of adenylate cyclase, breakdown of phosphoinositides and modulation of potassium channels through the action of G proteins. Primary transducing effect is adenylate cyclase inhibition. Polymorphism associated with IQ [60].
CTSD	cathepsin D (lysosomal aspartyl protease). Acid protease active in intracellular protein breakdown. Polymorphism associated with AD [61] and general intelligence in a healthy older population [62].
VEGF	vascular endothelial growth factor. Growth factor active in angiogenesis, vasculogenesis and endothelial cell growth. VEGF links hippocampal activity with neurogenesis, learning and memory [72].
	
**genes associated with AD**	
AGER	advanced glycosylation end product-specific receptor (RAGE). Mediates interactions of advanced glycosylation end products (AGE). Increased expression in AD [73].
APP	amyloid beta (A4) precursor protein. Polymorphisms associated with AD (reviewed in [34]).
HTR2A	5-hydroxytryptamine (serotonin) receptor 2A. This is one of the several different receptors for 5-hydroxytryptamine (serotonin), a biogenic hormone that functions as a neurotransmitter, a hormone, and a mitogen. Polymorphisms associated with episodic memory [63,74] and neuropsychiatric symptoms in AD [64].
IDE	insulin degrading enzyme. May play a role in the cellular processing of insulin. May be involved in intercellular peptide signaling. Polymorphism associated with AD [65].
IL1B	interleukin 1, beta. Produced by activated macrophages. IL-1 proteins are involved in the inflammatory response, being identified as endogenous pyrogens, and are reported to stimulate the release of prostaglandin and collagenase from synovial cells. Polymorphism associated with AD [66].
PLAU	plasminogen activator, urokinase. Polymorphisms associated with AD [68].
	
**stress response genes altered in aged mouse brain [28].**	
APOD	apolipoprotein D. APOD occurs in the macromolecular complex with lecithin-cholesterol acyltransferase. It is probably involved in the transport and binding of bilin. Appears to be able to transport a variety of ligands in a number of different contexts.
CRYAB	alpha B2 crystallin. May contribute to the transparency and refractive index of the lens.
CSNK1D	casein-kinase 1 delta. Casein kinases are operationally defined by their preferential utilization of acidic proteins such as caseins as substrates. It can phosphorylate a large number of proteins. Participates in Wnt signaling.
CTNNB1	catenin (cadherin-associated protein), beta 1, 88 kDa. Involved in the regulation of cell adhesion and in signal transduction through the Wnt pathway.
CTSD	cathepsin D. Acid protease active in intracellular protein breakdown. Involved in the pathogenesis of several diseases such as breast cancer and possibly Alzheimer's disease.
CTSH	cathespin H. Important for the overall degradation of proteins in lysosomes.
CTSS	cathespin S. Thiol protease. The bond-specificity of this proteinase is in part similar to the specificities of cathepsin L and cathepsin N.
CTSZ	cathepsin Z. Exhibits carboxy-monopeptidase as well as carboxy-dipeptidase activity.
DDIT3	gadd153 DNA-damage inducible transcript 3. Inhibits the DNA-binding activity of C/EBP and LAP by forming heterodimers that cannot bind DNA.
DNAJB1	DnaJ (Hsp40) homolog, subfamily B, member 1. Interacts with HSP70 and can stimulate its ATPase activity. Stimulates the association between HSC70 and HIP.
DNAJB2	DnaJ (Hsp40) homolog, subfamily B, member 2.
FOSB	FBJ murine osteosarcoma viral oncogene homolog B. FosB interacts with Jun proteins enhancing their DNA binding activity.
GFAP	glial fibrillary acidic protein. A class-III intermediate filament, is a cell-specific marker that, during the development of the central nervous system, distinguishes astrocytes from other glial cells.
JUNB	jun B proto-oncogene. Transcription factor involved in regulating gene activity following the primary growth factor response. Binds to the DNA sequence 5'-TGA [CG]TCA-3'.
NDRG1	N-myc downstream regulated gene 1. Cycophilin C associated protein. May have a growth inhibitory role.
NR2C2	nuclear receptor subfamily 2, group C, member 2. Orphan nuclear receptor. May regulate gene expression during the late phase of spermatogenesis.
SAA2	serum amyloid A2.
UCHL1	ubiquitin carboxyl-terminal esterase L1 (ubiquitin thiolesterase). Ubiquitin-protein hydrolase is involved both in the processing of ubiquitin precursors and of ubiquinated proteins. This enzyme is a thiol protease that recognizes and hydrolyzes a peptide bond at the C-terminal glycine of ubiquitin.
VIM	vimentin. Vimentins are class-III intermediate filaments found in various non-epithelial cells, especially mesenchymal cells.
	
**Mitochondria complex 1**	
NDUFA1	
NDUFA2	
NDUFA3	
NDUFA4	
NDUFA5	
NDUFA6	
NDUFA7	
NDUFA8	
NDUFA9	
NDUFA10	
NDUFAB1	
NDUFB1	
NDUFB2	
NDUFB3	
NDUFB4	
NDUFB5	
NDUFB6	
NDUFB7	
NDUFB8	
NDUFB9	
NDUFB10	
NDUFC1	
NDUFC2	
NDUFS1	
NDUFS2	
NDUFS3	
NDUFS4	
NDUFS5	
NDUFS6	
NDUFS7	
NDUFS8	
NDUFV1	
NDUFV2	
NDUFV3	

#### Genotyping of LBC1921

Genomic DNA was extracted from blood using standard methods. Genotyping of 384 SNPs was performed using the GoldenGate™ assay by the Illumina BeadLab service facility in San Diego. 444 LBC1921 subjects were genotyped, 16 of them in duplicate. A further three SNPs (*MPO*, rs7208693; *TF*, rs3811656 and *NDUFAF1 *rs3204853) were genotyped at the Welcome Trust Clinical Research Facility Genetics Core, Western General Hospital, Edinburgh[58] using TaqMan^® ^technology (Applied Biosystems).

#### Genotyping of ABC1936

Genomic DNA was extracted from blood using standard methods. Genotyping in LBC1921 found seven independent SNPs significantly associated with cognitive ageing (*p *< 0.01), and three SNPs significantly associated with age 11 MHT score. Genotyping for these SNPs was attempted in ABC1936, using KASPar, by Kbiosciences (Herts, UK). In cases where a KBiosciences assay could not be designed, genotyping was performed at the Welcome Trust Clinical Research Facility Genetics Core, Western General Hospital, Edinburgh[58] using TaqMan^® ^technology.

### Statistical analysis

The power to detect a causative variant at a type-1 error rate of 0.01, for a variant explaining 2–3% of the variance, was estimated by calculating the non-centrality parameter of a non-central χ^2 ^and the probability that the test statistic under the alternative hypothesis would be larger than the threshold corresponding to the specified type-1 error[59].

The effect of each SNP genotype on LBC1921 age 11 MHT score was analysed using general linear modelling (univariate analysis of variance). The fixed effects (between subjects variables) were: SNP genotype and sex.

The effect of each SNP genotype on each of the four age 79 cognitive outcome variables, for LBC1921, was analysed using general linear modelling (multivariate analysis of variance). The fixed effects were: SNP genotype and sex. Age 11 MHT score was included as a covariate, allowing us to identify associations specifically with cognitive ageing.

General linear modelling, as described above, was used to identify associations between SNPs that showed a positive association in LBC1921 (with either age 11 MHT score or cognitive ageing), and age 11 MHT score and each of the five age 64 cognitive outcome variables (controlling for age 11 MHT score), for ABC1936.

The raw data from LBC1921 and ABC1936 were combined and the effect of each SNP on the Raven's Progressive Matrices Score was analysed using general linear modelling (univariate analysis of variance). In addition to SNP genotype and sex, cohort was added to the model as a fixed effect and age 11 MHT score was included as a covariate.

All general linear modelling was performed using SPSS v12.0. Statistical significance was set at *p *< 0.01 for all statistical tests.

## Authors' contributions

IJD, AFW, LJW and JMS originally designed the study. All authors contributed to the development of the study design and helped draft the manuscript. All authors read and approved the final manuscript. SEH curated the LBC1921 database, performed the statistical analysis and drafted the manuscript. HF curated the ABC1936 database.

## Supplementary Material

Additional file 1 384 SNPs selected for genotyping by Illumina. The table provided lists the 384 SNPs that were submitted to Illumina for genotyping. Predicted SNP function, amino acid substitution (where relevant), percentage identity in mouse, and gene and chromosome locations are given for each SNP. Click here for file
