# Supplementary material for: A genetic association analysis of cognitive ability and cognitive ageing using 325 markers for 109 genes associated with oxidative stress or cognition
Source: BMC Genet. 2007 Jul 2;8:43. doi: 10.1186/1471-2156-8-43 (PMC1933580; doi:10.1186/1471-2156-8-43)
Supplement: Additional file 1 — 384 SNPs selected for genotyping by Illumina. The table provided lists the 384 SNPs that were submitted to Illumina for genotyping. Predicted SNP function, amino acid substitution (where relevant), percentage identity in mouse, and gene and chromosome locations are given for each SNP. [file 1471-2156-8-43-S1.doc]

**Additional file 1** 384 SNPs selected for genotyping by Illumina.

| **SNP Name** | **SNP function** | **Amino Acid substitution** | **Percentage identity in mouse** | **Gene** | **Chromosome** |
| --- | --- | --- | --- | --- | --- |
| rs3134943 | intron |  | 0.82 | AGER | 6 |
| rs1062070 | synonymous |  | 0.87 | AGER | 6 |
| rs2071288 | intron |  | 0.84 | AGER | 6 |
| rs2070600 | nonsynonymous | G68S | 0.88 | AGER | 6 |
| rs1800684 | synonymous |  | 0.84 | AGER | 6 |
| rs6786696 | 5' flanking (more than 2 kb) |  | 0.75 | APOD | 3 |
| rs17033096 | 5' flanking (more than 2 kb) |  | 0.88 | APOD | 3 |
| rs4686327 | 5' flanking (more than 2 kb) |  | 0.66 | APOD | 3 |
| rs1800557 | nonsynonymous | A713V | 0.92 | APP | 21 |
| rs1787439 | intron |  | 0.89 | APP | 21 |
| rs7278851 | intron |  | 0.75 | APP | 21 |
| rs17001582 | intron |  | 0.69 | APP | 21 |
| rs2040276 | intron |  | 0.83 | APP | 21 |
| rs2026225 | intron |  | 0.69 | APP | 21 |
| rs2830019 | intron |  | 0.87 | APP | 21 |
| rs2830020 | intron |  | 0.81 | APP | 21 |
| rs2830038 | intron |  | 0.8 | APP | 21 |
| rs1041420 | intron |  | 0.84 | APP | 21 |
| rs2830045 | intron |  | 0.75 | APP | 21 |
| rs2830048 | intron |  | 0.76 | APP | 21 |
| rs2830052 | intron |  | 0.85 | APP | 21 |
| rs3787650 | intron |  | 0.68 | APP | 21 |
| rs2830071 | intron |  | 0.78 | APP | 21 |
| rs9305282 | intron |  | 0.83 | APP | 21 |
| rs2830102 | intron |  | 0.87 | APP | 21 |
| rs9332971 | nonsynonymous | H856R |  | AR | X |
| rs481285 | 3' UTR |  | 0.78 | BACE | 11 |
| rs535860 | 3' UTR |  | 0.83 | BACE | 11 |
| rs539765 | nonsynonymous | C412R | 0.91 | BACE | 11 |
| rs638405 | synonymous |  | 0.91 | BACE | 11 |
| rs1001179 | functional promoter polymorphism[1]. |  |  | CAT | 11 |
| rs769217 | synonymous |  | 0.92 | CAT | 11 |
| rs11032709 | synonymous |  | 0.74 | CAT | 11 |
| rs234706 | synonymous |  | 0.87 | CBS$ | 21 |
| rs2298758 | synonymous |  | 0.89 | CBS$ | 21 |
| rs1110707 | nonsynonymous | A156T |  | CCS$ | 11 |
| rs3093728 | 5' UTR |  | 0.79 | CDKN1B | 12 |
| rs34330 | 5' UTR |  | 0.73 | CDKN1B | 12 |
| rs2066828 | nonsynonymous | R15W | 0.9 | CDKN1B | 12 |
| rs2066827 | nonsynonymous | V109G |  | CDKN1B | 12 |
| rs3093730 | intron |  | 0.77 | CDKN1B | 12 |
| rs4251698 | 3' UTR |  | 0.67 | CDKN1B | 12 |
| rs7330 | intron |  | 0.83 | CDKN1B | 12 |
| rs8191992 | 3'UTR associated with IQ[2]. |  |  | CHRM2 | 7 |
| rs16861582 | intron |  | 0.75 | CP | 3 |
| rs1523514 | intron |  | 0.73 | CP | 3 |
| rs1053709 | synonymous |  | 0.86 | CP | 3 |
| rs6799507 | intron |  | 0.83 | CP | 3 |
| rs701753 | nonsynonymous | E544D | 0.87 | CP | 3 |
| rs17838831 | 5' flanking |  | 0.84 | CP | 3 |
| rs11603779 | intron |  | 0.83 | CRYAB | 11 |
| rs4252581 | synonymous |  | 0.89 | CRYAB | 11 |
| rs14133 | 5' flanking |  | 0.9 | CRYAB | 11 |
| rs4252583 | 5' flanking |  | 0.82 | CRYAB | 11 |
| rs762550 | 3' flanking |  | 0.73 | CRYAB | 11 |
| rs6416862 | intron |  | 0.83 | CSNK1D | 17 |
| rs4135384 | nonsynonymous | M688V |  | CTNNB1 | 3 |
| rs1320294 | synonymous |  | 0.8 | CTSD | 11 |
| rs17571 | nonsynonymous associated with AD[3] and general intelligence [4]. | A58V | 0.71 | CTSD | 11 |
| rs13345 | synonymous |  | 0.81 | CTSH | 15 |
| rs12148472 | intron/splice site |  | 0.85 | CTSH | 15 |
| rs1036938 | nonsynonymous | C26S |  | CTSH | 15 |
| rs10888390 | nonsynonymous | W113R | 0.77 | CTSS | 1 |
| rs9760 | synonymous |  | 0.76 | CTSZ | 20 |
| rs2291136 | intron |  | 0.89 | DDIT3 | 12 |
| rs3962158 | synonymous |  | 0.89 | DNAJB1 | 19 |
| rs2276638 | intron |  | 0.88 | DNAJB2 | 2 |
| rs3731897 | intron |  | 0.78 | DNAJB2 | 2 |
| rs3821039 | intron |  | 0.83 | DNAJB2 | 2 |
| rs2282695 | synonymous |  | 0.9 | FOSB | 19 |
| rs2238686 | intron |  | 0.81 | FOSB | 19 |
| rs2883881 | intron |  | 0.86 | FOXO3A | 6 |
| rs17532874 | intron |  | 0.84 | FOXO3A | 6 |
| rs12202049 | intron |  | 0.86 | FOXO3A | 6 |
| rs12203787 | intron |  | 0.88 | FOXO3A | 6 |
| rs8108882 | synonymous |  | 0.88 | FTL | 19 |
| rs2066509 | synonymous |  | 0.81 | GCLC | 6 |
| rs2066510 | intron |  | 0.74 | GCLC | 6 |
| rs1555903 | intron |  | 0.92 | GCLC | 6 |
| rs3744473 | nonsynonymous | H364R | 0.75 | GFAP | 17 |
| rs3744470 | 3' UTR |  | 0.79 | GFAP | 17 |
| rs9916491 | intron |  | 0.86 | GFAP | 17 |
| rs1126642 | nonsynonymous | D295N | 0.89 | GFAP | 17 |
| rs2229012 | synonymous |  | 0.81 | GFAP | 17 |
| rs737128 | synonymous |  | 0.93 | GGT1 | 22 |
| rs4049829 | nonsynonymous | V272A | 0.82 | GGT1 | 22 |
| rs16986465 | nonsynonymous | A435V | 0.79 | GGT1 | 22 |
| rs4561 | synonymous |  | 0.81 | GLRX | 5 |
| rs3448 | 5' flanking |  | 0.87 | GPX1 | 3 |
| rs8178967 | nonsynonymous | S2N | 0.84 | GPX4 | 19 |
| rs4851 | 3' UTR |  |  | GPX4 | 19 |
| rs2020916 | nonsynonymous | P271H | 0.75 | GSR | 8 |
| rs8190976 | nonsynonymous | G189S | 0.82 | GSR | 8 |
| rs8190955 | nonsynonymous | R110C | 0.82 | GSR | 8 |
| rs8190895 | intron |  | 0.74 | GSR | 8 |
| rs2251780 | intron |  | 0.89 | GSR | 8 |
| rs6119545 | intron |  | 0.72 | GSS | 20 |
| rs7265992 | intron |  | 0.78 | GSS | 20 |
| rs2025096 | 5' UTR |  | 0.7 | GSS | 20 |
| rs1051866 | synonymous |  | 0.73 | GSTA1 | 6 |
| rs1051578 | nonsynonymous | T19I | 0.79 | GSTA1 | 6 |
| rs6577 | nonsynonymous | E210A | 0.74 | GSTA2 | 6 |
| rs2266631 | nonsynonymous | V149A | 0.83 | GSTA2 | 6 |
| rs2180314 | nonsynonymous | S112T | 0.61 | GSTA2 | 6 |
| rs1052661 | nonsynonymous | I71L | 0.9 | GSTA3 | 6 |
| rs1802061 | synonymous |  |  | GSTA4 | 6 |
| rs2397118 | nonsynonymous | V55I | 0.86 | GSTA5 | 6 |
| rs7803893 | intron |  | 0.89 | GSTK1 | 7 |
| rs1065411 | nonsynonymous | K173N | 0.84 | GSTM1 | 1 |
| rs7483 | nonsynonymous | V224I | 0.81 | GSTM3 | 1 |
| rs560018 | intron |  | 0.73 | GSTM4 | 1 |
| rs650985 | intron |  | 0.78 | GSTM4 | 1 |
| rs1051113 | nonsynonymous | V212M | 0.82 | GSTM4 | 1 |
| rs7536162 | intron |  | 0.76 | GSTM5 | 1 |
| rs17596954 | synonymous |  | 0.83 | GSTM5 | 1 |
| rs4925 | nonsynonymous | A140D | 0.65 | GSTO1 | 10 |
| rs11509438 | nonsynonymous | E208K | 0.65 | GSTO1 | 10 |
| rs156697 | nonsynonymous | N142D | 0.8 | GSTO2 | 10 |
| rs3758572 | synonymous |  | 0.8 | GSTO2 | 10 |
| rs8191448 | synonymous |  | 0.81 | GSTP1 | 11 |
| rs762803 | intron |  | 0.77 | GSTP1 | 11 |
| rs947894 | nonsynonymous | V105I | 0.84 | GSTP1 | 11 |
| rs1799811 | nonsynonymous | V114A | 0.88 | GSTP1 | 11 |
| rs1871042 | intron |  | 0.78 | GSTP1 | 11 |
| rs2266637 | nonsynonymous | V169I |  | GSTT1 | 22 |
| rs2266633 | nonsynonymous | D141N |  | GSTT1 | 22 |
| rs140188 | intron |  |  | GSTT2 | 22 |
| rs8177536 | nonsynonymous | A54E | 0.83 | GSTZ1 | 14 |
| rs2270421 | 5' flanking |  | 0.7 | GSTZ1 | 14 |
| rs2287395 | 5' UTR |  | 0.79 | GSTZ1 | 14 |
| rs3177429 | nonsynonymous | R42G | 0.83 | GSTZ1 | 14 |
| rs2287396 | intron |  | 0.82 | GSTZ1 | 14 |
| rs1046428 | nonsynonymous | M82T | 0.82 | GSTZ1 | 14 |
| rs2234955 | nonsynonymous | N133H | 0.78 | GSTZ1 | 14 |
| rs9282702 | nonsynonymous | L106P |  | HMOX1 | 22 |
| rs5755713 | nonsynonymous | Q152H |  | HMOX1 | 22 |
| rs6500610 | intron |  | 0.87 | HMOX2 | 16 |
| rs11643057 | intron |  | 0.74 | HMOX2 | 16 |
| rs17137094 | synonymous |  | 0.92 | HMOX2 | 16 |
| rs1665659 | intron |  | 0.77 | HSPA12A | 10 |
| rs2907235 | intron |  | 0.78 | HSPA12A | 10 |
| rs4752003 | intron |  | 0.86 | HSPA12A | 10 |
| rs1665638 | intron |  | 0.89 | HSPA12A | 10 |
| rs740599 | intron |  | 0.78 | HSPA12A | 10 |
| rs1638409 | intron |  | 0.73 | HSPA12A | 10 |
| rs1900501 | 5' flanking |  | 0.91 | HSPA12A | 10 |
| rs6052048 | synonymous |  | 0.84 | HSPA12B | 20 |
| rs3827077 | intron |  | 0.85 | HSPA12B | 20 |
| rs6076550 | synonymous |  | 0.81 | HSPA12B | 20 |
| rs6139194 | nonsynonymous | R270H | 0.84 | HSPA12B | 20 |
| rs2295340 | nonsynonymous | T115A |  | HSPA12B | 20 |
| rs506770 | synonymous |  | 0.92 | HSPA1A | 6 |
| rs2075800 | nonsynonymous | E602K | 0.84 | HSPA1L | 6 |
| rs2227956 | nonsynonymous | M493T | 0.88 | HSPA1L | 6 |
| rs2075799 | synonymous |  | 0.85 | HSPA1L | 6 |
| rs17101915 | 5' flanking |  | 0.78 | HSPA2 | 14 |
| rs3213995 | 3' UTR |  | 0.83 | HSPA2 | 14 |
| rs3213996 | 3' UTR |  | 0.78 | HSPA2 | 14 |
| rs11848114 | 3' flanking |  | 0.78 | HSPA2 | 14 |
| rs398606 | 3' flanking |  | 0.89 | HSPA4 | 5 |
| rs14355 | 3' UTR |  | 0.81 | HSPA4 | 5 |
| rs15233 | nonsynonymous | M333I |  | HSPA5 | 9 |
| rs7922 | 5' flanking |  | 0.93 | HSPA5 | 9 |
| rs430397 | intron |  | 0.78 | HSPA5 | 9 |
| rs400835 | nonsynonymous | R95Q |  | HSPA6 | 1 |
| rs10919226 | nonsynonymous | D154N |  | HSPA6 | 1 |
| rs386730 | nonsynonymous | T297K |  | HSPA6 | 1 |
| rs417707 | nonsynonymous | V336F |  | HSPA6 | 1 |
| rs9659608 | nonsynonymous | A375S |  | HSPA6 | 1 |
| rs407664 | nonsynonymous | R471H |  | HSPA6 | 1 |
| rs3820450 | nonsynonymous | E532K |  | HSPA6 | 1 |
| rs368844 | nonsynonymous | R577Q |  | HSPA6 | 1 |
| rs3763897 | intron |  | 0.87 | HSPA8 | 11 |
| rs1064585 | synonymous |  | 0.85 | HSPA8 | 11 |
| rs2236658 | 5' flanking |  | 0.84 | HSPA8 | 11 |
| rs10117 | synonymous |  |  | HSPA9B | 5 |
| rs17069218 | 3' UTR |  | 0.86 | HTR2A | 13 |
| rs3803189 | 3' UTR |  | 0.83 | HTR2A | 13 |
| rs6314 | nonsynonymous associated with episodic memory[5]. | H452Y | 0.78 | HTR2A | 13 |
| rs1058576 | nonsynonymous | S421F |  | HTR2A | 13 |
| rs1923884 | intron |  | 0.72 | HTR2A | 13 |
| rs6305 | synonymous |  | 0.87 | HTR2A | 13 |
| rs6313 | synonymous associated with AD[6]. |  |  | HTR2A | 13 |
| rs6316 | 5' flanking |  | 0.86 | HTR2A | 13 |
| rs9595555 | 5' flanking (more than 2 kb) |  | 0.76 | HTR2A | 13 |
| rs12356364 | nonsynonymous | D947N | 0.9 | IDE | 10 |
| rs7895832 | intron |  | 0.77 | IDE | 10 |
| rs3758505 | 5' flanking (more than 2 kb) associated with AD[7]. |  |  | IDE | 10 |
| rs1143634 | synonymous associated with AD[8]. |  | 0.69 | IL1B | 2 |
| rs16062 | synonymous |  | 0.72 | IL1B | 2 |
| rs1143627 | 5' flanking |  | 0.64 | IL1B | 2 |
| rs1061595 | 3' UTR |  | 0.88 | JUNB | 19 |
| rs4683233 | nonsynonymous | A29T |  | LTF | 3 |
| rs2759 | nonsynonymous | I717V |  | MPO | 17 |
| rs7208693 | nonsynonymous | V53F |  | MPO | 17 |
| rs12679328 | intron |  | 0.81 | MSRA | 8 |
| rs3735823 | intron |  | 0.75 | MSRA | 8 |
| rs814422 | intron |  | 0.73 | MSRA | 8 |
| rs17151064 | intron |  | 0.8 | MSRA | 8 |
| rs10503401 | intron |  | 0.81 | MSRA | 8 |
| rs1994224 | intron |  | 0.86 | MSRA | 8 |
| rs6601414 | intron |  | 0.81 | MSRA | 8 |
| rs17151140 | intron |  | 0.84 | MSRA | 8 |
| rs17151158 | intron |  | 0.71 | MSRA | 8 |
| rs17151175 | intron |  | 0.71 | MSRA | 8 |
| rs1484645 | intron |  | 0.85 | MSRA | 8 |
| rs6986977 | intron |  | 0.94 | MSRA | 8 |
| rs877390 | intron |  | 0.74 | MSRA | 8 |
| rs7845503 | intron |  | 0.77 | MSRA | 8 |
| rs6992349 | intron |  | 0.75 | MSRA | 8 |
| rs4288376 | intron |  | 0.71 | MSRA | 8 |
| rs10503405 | intron |  | 0.78 | MSRA | 8 |
| rs6983870 | intron |  | 0.83 | MSRA | 8 |
| rs4260895 | intron |  | 0.7 | MSRA | 8 |
| rs2952182 | intron |  | 0.85 | MSRA | 8 |
| rs11783281 | intron |  | 0.75 | MSRA | 8 |
| rs17151588 | intron |  | 0.82 | MSRA | 8 |
| rs6983361 | intron |  | 0.86 | MSRA | 8 |
| rs7832708 | intron |  | 0.82 | MSRA | 8 |
| rs11781529 | intron |  | 0.86 | MSRA | 8 |
| rs4841322 | intron |  | 0.77 | MSRA | 8 |
| rs4841324 | intron |  | 0.79 | MSRA | 8 |
| rs17151867 | intron |  | 0.74 | MSRA | 8 |
| rs3750314 | synonymous |  | 0.83 | MSRA | 8 |
| rs1555804 | intron |  | 0.75 | MSRB | 10 |
| rs10764383 | intron |  | 0.87 | MSRB | 10 |
| rs11013295 | intron |  | 0.82 | MSRB | 10 |
| rs7427 | synonymous |  | 0.82 | MSRB | 10 |
| rs2977499 | intron |  | 0.75 | NDRG1 | 8 |
| rs2272653 | intron |  | 0.84 | NDRG1 | 8 |
| rs2930002 | intron |  | 0.82 | NDRG1 | 8 |
| rs2083411 | synonymous |  | 0.73 | NDUFA10 | 2 |
| rs254259 | intron |  | 0.82 | NDUFA3 | 19 |
| rs8139803 | synonymous |  | 0.91 | NDUFA6 | 22 |
| rs1801311 | nonsynonymous | A9V |  | NDUFA6 | 22 |
| rs561 | synonymous |  | 0.84 | NDUFA7 | 19 |
| rs2288415 | nonsynonymous | P70A | 0.85 | NDUFA7 | 19 |
| rs2241591 | 5' flanking |  | 0.86 | NDUFA7 | 19 |
| rs4147659 | 3' flanking |  | 0.66 | NDUFA8 | 9 |
| rs6822 | 3' UTR |  | 0.81 | NDUFA8 | 9 |
| rs4679 | synonymous |  | 0.83 | NDUFA8 | 9 |
| rs4147672 | intron |  | 0.75 | NDUFA9 | 12 |
| rs4147682 | intron |  | 0.96 | NDUFA9 | 12 |
| rs459894 | intron |  |  | NDUFAB1 | 16 |
| rs2302175 | synonymous |  | 0.84 | NDUFB10 | 16 |
| rs2339844 | nonsynonymous | C10G |  | NDUFB5 | 3 |
| rs3817369 | 5' UTR |  | 0.72 | NDUFB5 | 3 |
| rs3752220 | nonsynonymous | R106G | 0.72 | NDUFB7 | 19 |
| rs9543 | synonymous |  | 0.84 | NDUFB7 | 19 |
| rs12259919 | intron |  | 0.75 | NDUFB8 | 10 |
| rs1802224 | nonsynonymous | S125P | 0.81 | NDUFB8 | 10 |
| rs1800662 | intron |  | 0.71 | NDUFB8 | 10 |
| rs11547284 | nonsynonymous | S146P | 0.85 | NDUFB9 | 8 |
| rs8875 | nonsynonymous | L46V | 0.77 | NDUFC2 | 11 |
| rs6435328 | intron |  | 0.86 | NDUFS1 | 2 |
| rs11548670 | synonymous |  | 0.91 | NDUFS1 | 2 |
| rs4147707 | 5' UTR |  | 0.65 | NDUFS1 | 2 |
| rs3813624 | intron |  | 0.8 | NDUFS2 | 1 |
| rs16832694 | intron |  | 0.79 | NDUFS2 | 1 |
| rs16827493 | nonsynonymous | A229P | 0.88 | NDUFS2 | 1 |
| rs16832699 | synonymous |  | 0.9 | NDUFS2 | 1 |
| rs11587213 | 3' flanking |  | 0.84 | NDUFS2 | 1 |
| rs2233358 | intron |  | 0.63 | NDUFS3 | 11 |
| rs3740654 | synonymous |  | 0.89 | NDUFS3 | 11 |
| rs4147732 | 5' flanking |  | 0.82 | NDUFS4 | 5 |
| rs2279516 | synonymous |  | 0.83 | NDUFS4 | 5 |
| rs13156337 | intron |  | 0.89 | NDUFS4 | 5 |
| rs31304 | synonymous |  | 0.92 | NDUFS4 | 5 |
| rs31303 | synonymous |  | 0.98 | NDUFS4 | 5 |
| rs567 | intron |  | 0.88 | NDUFS4 | 5 |
| rs1050978 | synonymous |  | 0.71 | NDUFS5 | 1 |
| rs3776141 | intron |  | 0.74 | NDUFS6 | 5 |
| rs1142530 | nonsynonymous | P23L |  | NDUFS7 | 19 |
| rs3133266 | intron |  | 0.75 | NDUFS8 | 11 |
| rs3115545 | intron |  | 0.74 | NDUFS8 | 11 |
| rs11227859 | synonymous |  | 0.89 | NDUFV1 | 11 |
| rs12283777 | nonsynonymous | I408T | 0.78 | NDUFV1 | 11 |
| rs906807 | nonsynonymous | V29A | 0.91 | NDUFV2 | 18 |
| rs4148973 | synonymous |  | 0.7 | NDUFV3 | 21 |
| rs8128440 | nonsynonymous | N398D |  | NDUFV3 | 21 |
| rs2293044 | synonymous |  | 0.87 | NOS1 | 12 |
| rs9658501 | synonymous |  | 0.87 | NOS1 | 12 |
| rs3741475 | synonymous |  | 0.89 | NOS1 | 12 |
| rs9658481 | intron |  | 0.89 | NOS1 | 12 |
| rs10774909 | intron |  | 0.8 | NOS1 | 12 |
| rs2291906 | synonymous |  | 0.9 | NOS1 | 12 |
| rs9658446 | synonymous |  | 0.91 | NOS1 | 12 |
| rs2293054 | synonymous |  | 0.87 | NOS1 | 12 |
| rs11612772 | intron |  | 0.82 | NOS1 | 12 |
| rs561712 | intron |  | 0.79 | NOS1 | 12 |
| rs3741481 | 5' UTR |  | 0.83 | NOS1 | 12 |
| rs9658256 | 5' UTR |  | 0.91 | NOS1 | 12 |
| rs1060826 | synonymous |  | 0.85 | NOS2A | 17 |
| rs2297512 | intron |  | 0.84 | NOS2A | 17 |
| rs2297518 | nonsynonymous | S608L |  | NOS2A | 17 |
| rs1137933 | synonymous |  | 0.79 | NOS2A | 17 |
| rs3730017 | nonsynonymous | R221W | 0.82 | NOS2A | 17 |
| rs3730014 | synonymous |  | 0.72 | NOS2A | 17 |
| rs2290510 | coding exon |  | 0.79 | NOS2B | 17 |
| rs3918159 | intron |  | 0.84 | NOS3 | 7 |
| rs3918162 | 5' UTR |  | 0.79 | NOS3 | 7 |
| rs3918166 | nonsynonymous | R112Q | 0.81 | NOS3 | 7 |
| rs1549758 | synonymous |  | 0.9 | NOS3 | 7 |
| rs1799983 | nonsynonymous associated with mild cognitive impairment[9]. | D298E |  | NOS3 | 7 |
| rs2566514 | synonymous |  | 0.91 | NOS3 | 7 |
| rs3918232 | nonsynonymous | V827M | 0.84 | NOS3 | 7 |
| rs3918201 | nonsynonymous | R885M | 0.85 | NOS3 | 7 |
| rs867225 | intron |  | 0.72 | NOS3 | 7 |
| rs3918234 | nonsynonymous | Q982L | 0.87 | NOS3 | 7 |
| rs1065300 | intron |  | 0.88 | NOS3 | 7 |
| rs3730011 | synonymous |  | 0.91 | NOS3 | 7 |
| rs3918211 | synonymous |  | 0.84 | NOS3 | 7 |
| rs17040625 | 5' UTR |  | 0.87 | NR2C2 | 3 |
| rs7617704 | nonsynonymous | F36L |  | NR2C2 | 3 |
| rs17040681 | synonymous |  | 0.96 | NR2C2 | 3 |
| rs17536979 | synonymous |  | 0.88 | NR2C2 | 3 |
| rs648912 | 3' flanking |  | 0.9 | NR2C2 | 3 |
| rs2227564 | nonsynonymous associated with AD[10]. | L141P | 0.84 | PLAU | 10 |
| rs2227567 | nonsynonymous | K231Q | 0.74 | PLAU | 10 |
| rs2227568 | synonymous |  | 0.74 | PLAU | 10 |
| rs1050122 | synonymous |  | 0.79 | PLAU | 10 |
| rs3805118 | nonsynonymous | R398L | 0.77 | PLAU | 10 |
| rs4065 | 3' UTR |  | 0.75 | PLAU | 10 |
| rs6954345 | nonsynonymous | S311C | 0.82 | PON2 | 7 |
| rs10487133 | intron |  | 0.84 | PON2 | 7 |
| rs11545941 | nonsynonymous | A148G | 0.91 | PON2 | 7 |
| rs17166875 | intron |  | 0.77 | PON2 | 7 |
| rs3779492 | intron |  | 0.69 | PON2 | 7 |
| rs6667191 | intron |  | 0.83 | PRDX1 | 1 |
| rs3790584 | 5' flanking |  | 0.69 | PRDX1 | 1 |
| rs10413408 | intron |  | 0.83 | PRDX2 | 19 |
| rs10422248 | intron |  | 0.74 | PRDX2 | 19 |
| rs3758609 | synonymous |  | 0.87 | PRDX3 | 10 |
| rs513573 | intron |  | 0.85 | PRDX4 | X |
| rs552105 | intron |  | 0.79 | PRDX4 | X |
| rs1548734 | intron |  | 0.66 | PRDX4 | X |
| rs7938623 | nonsynonymous | Y33C |  | PRDX5 | 11 |
| rs2468844 | nonsynonymous | R89H |  | SAA2 | 11 |
| rs4987017 | 3' UTR |  | 0.76 | SEPP1 | 5 |
| rs6413428 | 3' UTR |  | 0.82 | SEPP1 | 5 |
| rs7579 | 3' flanking |  | 0.79 | SEPP1 | 5 |
| rs2273773 | synonymous |  | 0.87 | SIRT1. | 10 |
| rs2234975 | 3' UTR |  | 0.94 | SIRT1. | 10 |
| rs9369628 | intron |  | 0.89 | SLC25A27 | 6 |
| rs12192544 | nonsynonymous | R23P |  | SLC25A27 | 6 |
| rs3757241 | synonymous |  | 0.89 | SLC25A27 | 6 |
| rs5746129 | nonsynonymous | R156W | 0.83 | SOD2 | 6 |
| rs4987023 | nonsynonymous | G76R | 0.94 | SOD2 | 6 |
| rs1804451 | synonymous |  | 0.95 | SOD2 | 6 |
| rs1799725 | nonsynonymous | V16A |  | SOD2 | 6 |
| rs2536512 | nonsynonymous | A58T |  | SOD3 | 4 |
| rs1799895 | nonsynonymous | R231G | 0.75 | SOD3 | 4 |
| rs1130459 | 5' UTR |  | 0.89 | TF | 3 |
| rs1799852 | synonymous |  | 0.79 | TF | 3 |
| rs1799899 | nonsynonymous | G277S | 0.76 | TF | 3 |
| rs8177238 | nonsynonymous | D296G | 0.86 | TF | 3 |
| rs2692696 | nonsynonymous | I448V | 0.77 | TF | 3 |
| rs8177286 | synonymous |  | 0.81 | TF | 3 |
| rs1049296 | nonsynonymous | P589S | 0.8 | TF | 3 |
| rs4135162 | intron |  | 0.83 | TXN | 9 |
| rs2281082 | intron |  | 0.88 | TXN2 | 22 |
| rs11111979 | 5' UTR |  | 0.8 | TXNRD1 | 12 |
| rs7134193 | 5' UTR |  | 0.75 | TXNRD1 | 12 |
| rs4964287 | synonymous |  | 0.88 | TXNRD1 | 12 |
| rs1127953 | nonsynonymous | R156S | 0.85 | TXNRD1 | 12 |
| rs3827288 | intron |  | 0.87 | TXNRD2 | 22 |
| rs5992495 | nonsynonymous | S299R | 0.8 | TXNRD2 | 22 |
| rs3747068 | synonymous |  | 0.84 | TXNRD2 | 22 |
| rs5748469 | nonsynonymous | A36S | 0.86 | TXNRD2 | 22 |
| rs5746847 | intron |  | 0.74 | TXNRD2 | 22 |
| rs777241 | synonymous |  | 0.88 | TXNRD3 | 3 |
| rs3732532 | synonymous |  | 0.89 | TXNRD3 | 3 |
| rs660339 | nonsynonymous | A55V | 0.82 | UCP2 | 11 |
| rs2010963 | 5' UTR |  | 0.91 | VEGF | 6 |
| rs833068 | intron |  | 0.71 | VEGF | 6 |
| rs3025000 | intron |  | 0.9 | VEGF | 6 |
| rs3025010 | intron |  | 0.69 | VEGF | 6 |
| rs3025024 | intron |  | 0.81 | VEGF | 6 |
| rs3025039 | 3' UTR |  | 0.88 | VEGF | 6 |
| rs3025040 | 3’ UTR |  | 0.76 | VEGF | 6 |
| rs10434 | 3' UTR |  | 0.72 | VEGF | 6 |
| rs3025053 | 3’ UTR |  | 0.78 | VEGF | 6 |
| rs3758410 | 5' UTR |  | 0.8 | VIM | 10 |
| rs1049341 | 3' UTR |  | 0.85 | VIM | 10 |

Amino acid substitutions for nonsynonymous SNPs are given. SNPs that have previously been associated with Alzheimer’s Disease (AD), cognitive ability or gene function are indicated. The percentage identity with mouse is based on a 120 base pair window surrounding the SNP. UTR = untranslated region. Flanking indicates that the SNP is within 2 kilobases of the gene unless otherwise indicated.

References

1. Forsberg L, Lyrenas L, de Faire U, Morgenstern R: **A common functional C-T substitution polymorphism in the promoter region of the human catalase gene influences transcription factor binding, reporter gene transcription and is correlated to blood catalase levels.** *Free Radic Biol Med* 2001, **30:** 500-505.

2. Comings DE, Wu S, Rostamkhani M, McGue M, Lacono WG, Cheng LS *et al*.: **Role of the cholinergic muscarinic 2 receptor (CHRM2) gene in cognition.** *Mol Psychiatry* 2003, **8:** 10-11.

3. Papassotiropoulos A, Bagli M, Feder O, Jessen F, Maier W, Rao ML *et al*.: **Genetic polymorphism of cathepsin D is strongly associated with the risk for developing sporadic Alzheimer's disease.** *Neurosci Lett* 1999, **262:** 171-174.

4. Payton A, Holland F, Diggle P, Rabbitt P, Horan M, Davidson Y *et al*.: **Cathepsin D exon 2 polymorphism associated with general intelligence in a healthy older population.** *Mol Psychiatry* 2003, **8:** 14-18.

5. de Quervain DJ, Henke K, Aerni A, Coluccia D, Wollmer MA, Hock C *et al*.: **A functional genetic variation of the 5-HT2a receptor affects human memory.** *Nat Neurosci* 2003, **6:** 1141-1142.

6. Lam LC, Tang NL, Ma SL, Zhang W, Chiu HF: **5-HT2A T102C receptor polymorphism and neuropsychiatric symptoms in Alzheimer's disease.** *Int J Geriatr Psychiatry* 2004, **19:** 523-526.

7. Edland SD, Wavrant-De Vriese F, Compton D, Smith GE, Ivnik R, Boeve BF *et al*.: **Insulin degrading enzyme (IDE) genetic variants and risk of Alzheimer's disease: evidence of effect modification by apolipoprotein E (APOE).** *Neurosci Lett* 2003, **345:** 21-24.

8. Sciacca FL, Ferri C, Licastro F, Veglia F, Biunno I, Gavazzi A *et al*.: **Interleukin-1B polymorphism is associated with age at onset of Alzheimer's disease.** *Neurobiol Aging* 2003, **24:** 927-931.

9. Sole-Padulles C, Bartres-Faz D, Junque C, Via M, Matarin M, Gonzalez-Perez E *et al*.: **Poorer cognitive performance in humans with mild cognitive impairment carrying the T variant of the Glu/Asp NOS3 polymorphism.** *Neurosci Lett* 2004, **358:** 5-8.

10. Ertekin-Taner N, Ronald J, Feuk L, Prince J, Tucker M, Younkin L *et al*.: **Elevated amyloid beta protein (Abeta42) and late onset Alzheimer's disease are associated with single nucleotide polymorphisms in the urokinase-type plasminogen activator gene.** *Hum Mol Genet* 2005, **14:** 447-460.
